# Knowledge, attitude and practices of buruli ulcer among residents in Jasikan municipality of Ghana: an ethnographic study

**DOI:** 10.1186/s12889-025-23367-y

**Published:** 2025-07-03

**Authors:** Atubiga Alobit Baba, Stanley Cowther, Michael Adjabeng, John Owusu Gyapong

**Affiliations:** 1 Tamale Technical University, P.O. Box 3 E/R, Tamale, Northern Region Ghana; 2 World Health Organization (WHO), Korle-Bu, Box KB 493, Accra, Ghana; 3 University of Health and Allied Sciences, PMB 31, Ho, Volta Region, Ghana

**Keywords:** Thematic, Buruli, Knowledge, Attitude, Practice

## Abstract

**Background:**

Buruli ulcer is part of the neglected tropical diseases in the world. The disease often starts with a pre-ulcerative nodule, a plaque or oedema which breaks down to form characteristic ulcers with undermined edges. The mode of transmission and identifying source reservoirs of the causative organism of the disease are still largely unknown.

**Purpose:**

This study explored the knowledge, attitude and practices of Buruli ulcer among residents in Jasikan Municipality of Ghana using an ethnographic study.

**Method:**

The study employed an ethnographic approach. The data were collected from 20 study participants using in-depth interview guide. The data were presented using thematic analysis.

**Results:**

Respondents *knowledge on Buruli ulcer varied. Some respondents attributed the cause of the disease to evil spirits and some respondents had no knowledge on what cause the disease.* The study found that Buruli ulcer patients sought treatment in *health facilities*, *prayer camps*, *herbalists home* and practiced *self-medications*. The findings revealed that, residents without the disease *had negative attitude towards Buruli ulcer patients*.

**Conclusions:**

Knowledge of respondents on Buruli ulcer varied and various practices were used to manage the disease. The attitude of participants towards the disease was unfavourvable.

**Supplementary Information:**

The online version contains supplementary material available at 10.1186/s12889-025-23367-y.

## Introduction

Buruli ulcer is a destructive skin and soft tissue infection caused by *Mycobacterium (M) ulcerans* [1]. Clinically, the disease starts with a papule, nodule, plaque, or edematous lesion that eventually progress to extensive skin ulceration. Because of the extent of tissue loss, the lesion is usually painless or only with limited pain [2]. The disease epidemiology is characterized by its patchy focal distribution within countries where it is endemic [3]. Persons living or working close to slow flowing or stagnant water bodies who fail to wear protective clothing, or living around low-lying marshes, wetlands, riverine areas and inappropriate care of skin wounds are at risk of being infected by the *M. ulcerans* [4].

However, the exact mode of the disease transmission and the environmental reservoir (s) of *M. ulcerans* remain mysterious [5], as culturing the slow-growing *M*. ulcerans from nonclinical environmental sources has proven to be particularly challenging. Despite that, Buruli ulcer cases have been reported in Sub-Saharan Africa especially in Cote d’Ivoire, Nigeria, Ghana and Benin among all age groups [1]. The disease is responsible for considerable suffering, functional disability (such as limited joint movement), particularly where children are victims, costly treatment and loss of productivity in the population, including deformity, bone infection and secondary bacterial infection of skin ulcer lesions [6].

In Ghana, the first probable case of Buruli ulcer was recorded in 1971 in a child who lived with the parents near the Densu River [28]. Following that case, in 1989, about 96 cases were reported in the Ashanti Region from the Afram Plains in the Asante Akim North District [29]. Between the period of 1993 and 1998 there were a number of Buruli ulcer cases recorded, up to 300 cases in the Amansie West District of the Ashanti Region [30]. These were among residents who lived near the Oda and Offin rivers. This suggested that, the people could have gotten the disease from the rivers at the time.

There is limited awareness of the disease both within the medical community and among the general public, resulting in under-recognition and under-reporting in Jasikan Municipality. The World Health Organization (WHO) recommends an eight-week course of antibiotics based on rifampicin, as well as adjuvant debridement and skin grafting if necessary [7].

While biomedical research on Buruli ulcer transmission takes environmental factors into consideration, residents’ knowledge within a geographic setting depends on their socio-cultural belief systems about the disease. This is because, societies knowledge about Buruli ulcer goes beyond ecological and biological aspects, and concerns all aspects of social life including religious beliefs about Buruli ulcer [8].

In other geographic settings, the basis of Buruli ulcer is considered as natural and mystical [9]. Therefore, a lack of information on the mode of transmission of Buruli ulcer, disease timeline and treatment outcomes may explain these interpretations linked to evil spirits and witchcraft [10]. Therefore, understanding local residents’ knowledge, attitude and practices in the context of Buruli ulcer in their natural setting would provide a better way to help policy makers implement health intervention strategies to help reduce the occurrence of the disease in the study setting. Since knowledge, attitude and practices of residents were not examined comprehensively among residents at the time of the study, there were limited information in that regards in empirical literature. Accordingly this study was conducted to examine the knowledge, attitude and practices of Buruli ulcer among residents in Jasikan Municipality of Ghana using an ethnographic study.

## Materials and methods

### Study setting

The study was conducted in the Jasikan Municipality. The Jasikan Municipality is one of the Municipalities in the Oti Region of Ghana. Majority of the population live in rural areas constituting about 72.4% [11]. There are health centres and hospital in the Municipality providing healthcare to the people around their catchment areas and beyond. The health centres are geographically located so as to provide easy access to health services to the people. The hospital also report cases of Buruli since it provide healthcare to residents. The terrain in the Municipality is generally undulating. The low-lying areas, some of which are swampy average 456.4 m above sea level, and are used for rice cultivation. The Jasikan Municipality was chosen for the study because of the rising cases of Buruli ulcer among the population. Available data confirmed that, as of 2018, 56 suspected cases of Buruli ulcer were reported in the Jasikan Municipality. In 2019, 2020 and 2021 recorded 1, 21 and 24 cases of Buruli ulcer respectively [11].

### Research approach

This study primarily used the ethnographic approach. The study employed this approach because ethnography considers the cultural orientation of the people’s way of life within their natural setting [12, 13]. The researchers lived among the community members with the view to understanding the culture and opinions they shared about the concept in this case, Buruli ulcer [13]. The study considered this approach very relevant to the study of Buruli ulcer because it enabled the study to gather the relevant data from residents about Buruli ulcer.

### Sampling method

The study employed purposive sampling method in the form of maximum variation to sample the respondents for the study. This sampling technique was chosen to gather data from both residents with the disease (Buruli ulcer and those without the disease). This provided the opportunity to collect diverge views from residents in terms of their knowledge, attitude and practices in relation to Buruli ulcer.

### Study participants and sample size

The study participants were residents living in the Jasikan Municipality at the time of the study. The study included residents of Jasikan Municipality who have stayed at least one year before the study and were confirmed cases of Buruli ulcer patients in the Jasikan Municipality. The study also included residents who did not have the Buruli ulcer disease. The study excluded residents with wounds who were seriously ill and needed serious health care. In terms of the sample size, 20 participants were sampled made up of 10 Buruli ulcer patients and 10 participants without Buruli ulcer. This sample size was justified because [14] revealed that, samples in qualitative research tend to be small in order to support the depth of case-oriented analysis that is fundamental to this mode of inquiry.

Additionally [15], posits that the more useable data are collected from each person, the fewer participants are needed in qualitative inquiry [16]. maintained that the experience of most qualitative researchers conducting an interview-based study with a fairly specific research question is that little new information is generated after interviewing 20 people or so belonging to one analytically relevant participant ‘category’ (pp. 102–104) [17]. indicated that data saturation is reached at the 17th interview for all their pre-determined theoretical constructs. Based on these arguments, because information rich participants were selected for the study, they had the capacity to provide richly-textured information, relevant to the phenomenon under investigation.

### In-depth interview guide

The study employed the use of in-depth interview (IDI) guide to collect the data from the respondents. This in-depth interview (IDI) guide was developed by the study and used for the data collection from both cases and controls by the help of trained health staff at the study setting. The interview was held at a quiet place to avoid unnecessary disruption that could affect the quality of the data. The used of IDI guide provided the opportunity for the study to collect detailed data from the various respondents on Buruli ulcer.

### Data collection

The data collection was supported by the Surveillance Officers at the Municipality. Data collection was done within one month period. Data collection began after the purpose of the study had been explained to participants. Participants consented to participate in the study. The interviews lasted for at least 30 min with each participant in the study setting. The interviews were conducted in a private place where participants were made comfortable enough to express themselves without any hindrance. Interviews were mainly conducted in English and the local languages that were preferred by participants. These enabled the study participants to express themselves very well in terms of their knowledge, attitude and practices on Buruli ulcer.

### Rigor

The study used confirmability, credibility, transferability and reflexivity to explain how the methodological rigor was achieved in the study. In terms of achieving confirmability, the study ensured that the interpretations of the data did not influence the researchers’ own prejudices, knowledge and past experiences with regards to Buruli ulcer. The researchers carried out the study in an orderly manner, keeping field notes to describe events and processes pertaining to data collection. To ensure credibility of the study, the period stated for the data collection which was at least 30 min enabled the research team to gather the relevant data from the respondents. Issue of transferability was handled explicitly. For instance, the study setting is known in Ghana as a Municipality in the Oti region. The procedure for gathering the data was also well described to enable any other person who wants to carry out the work employ similar methods for the study.

To address the issue of reflexivity, the research team approached this issue very carefully by examining their own conceptual understanding of the topic through a clear lens allowing study participants to explicitly and implicitly express their opinions on the subject matter freely. The values and preconceptions of the research team did not influence the decision of the data collection process and by extension all the phases of the study. The data was collected by trained health workers in the study setting.

### Ethical considerations

The study was performed in accordance with the ethical standards as laid down in the 1964 Declaration of Helsinki and its later amendments and its later amendments. The approval Committee was the University of Health and Allied Sciences (UHAS) Research Ethics Committee (REC). The approval number was (UHAS-REC A.11 [103] 2 L-22) as part of a study. Permission was sought from the Jasikan Municipality Health Directorate. A written informed consent from parents/guardians and ascent from children was also obtained. Informed consent to participate was obtained from all the study participants in the study setting. The participants’ information sheet explained the purpose, benefits and data collection procedures as well as possible risks, to the participants. Names and identifying information were all anonymized. There was voluntary participation of study participants. Additionally information concerning this study which was part of a broader is included in the manuscript.

### Data analysis

The data were uploaded onto a computer and transferred onto qualitative software NVivo 12, to support data coding and thematic analysis. Each transcript was checked for accuracy against the data. The researchers read several times to reach an overall understanding. Data were presented in a narrative form that described the various responses established in the coded thematic analyses. The coded responses were checked by experts to ensure that the recorded data matched with the transcribed data.

## Results

Table 1 shows the demographic characteristics of the participants. The study sample involved 20 participants of which majority of them were females.

**Table 1:** Demographic data of respondents

Age (years)	Cases (*n* = 20)
18–20	1
21–40	7
41+	12
**Sex**	
Male	6
Female	14
**Occupational status**	
Petty trading	4
Farming	6
Salaried worker	3
Unemployed	7
**Marital status**	
Single	2
Married	12
Separated	6
**Educational status**	
No formal education	2
Formal education	18

Major themes and sub themes that emerged from the data shown in Table 2.

**Table 2:** Themes from buruli ulcer patients responses

Main themes	Sub themes
**Knowledge of Buruli ulcer**	
Understanding of Buruli ulcer	Wound caused by the environment
	A wound that takes long time to heal
	Wound that starts small and becomes big
Signs and symptoms of Buruli ulcer	Wound that is not going
Natural wound	Wound that just come on its own
Painful wound	Wound that is painful and not healing
Wound cause by evil spirits	The wound is caused by evil spirits
Unknown cause	The cause of Buruli ulcer is not known
**Attitude towards Buruli ulcer**	
Less attention to wound due to cost	Managing the wound is too costly
No concern until it is serious	There is no seriousness of the disease until it becomes serious
Transportation cost is high	Going to health centre to dress the wound is too costly and expensive
Less socialization	Less participation in social activities
**Practices on Buruli ulcer**	
Managing Buruli ulcer	Use local herbs to manage the disease
	I go to health centre to dress the wound
Use counter medicine	Use counter medicines to manage the wound
Purchase of dressing materials	Dressing materials are purchased for use
Visitation of prayer camps	Visits prayer camps for healing
Herbal medicine	Use herbal medicine to treat the wound
Visit hospital	Visit the hospital for wound dressing

Table 3, presents themes from responses of participants without Buruli ulcer.

**Table 3:** Themes from residents without buruli ulcer responses

Main themes	Sub themes
**Knowledge of Buruli ulcer**	
Understanding of Buruli ulcer	Wound that is painful
	I have no idea about Buruli ulcer
**Attitude towards Buruli ulcer**	
Uncomfortable with the disease	Feel uncomfortable with the disease
Less favourable	I do not attend health centre regularly
	No concern until it is serious
	Managing the wound is too costly
**Practices on Buruli ulcer**	
Managing Buruli ulcer	Herbal medicines are good for the disease
	Encourage people to go to hospital
Move to prayer camps	Attend prayers camps for healing of the wound
Clinics for wound dressing	Some of the patients visit the clinic for wound dressing
Covering the wound	Always make sure the wound is covered
Application of herbal medicine	Cases applied herbal medicine on the wound

### Theme 1: knowledge of residents on buruli ulcer

#### Participants Understanding of buruli ulcer

The study participants were asked to explain how they understood Buruli ulcer. Based on the responses, study participants expressed various explanations to the disease. Excerpts are illustrated below.

- *Buruli ulcer is when a person has a wound that is caused by the environment the person is exposed to here in Jasikan Municipality…***Buruli ulcer patient** (**P: 1)**.

In this explanation by the study participant, the Buruli ulcer patient is basing the meaning of Buruli ulcer to the environment in the study area. The participant explanation could be linked to the environmental pathogen hypothesized to be the causative organism for the disease.

Another participant explained what Buruli ulcer is as;

- *Buruli ulcer is when a person has a wound and the wound takes a long time to heal in this place…***Buruli ulcer patient** (**P: 2)**.

The explanation of the disease attributed to the wound could be linked to the nature of how the patient had the wound for a long time and was hoping the wound would heal within the shortest possible time.

Another study participant explained the meaning of Buruli ulcer as;

- *Buruli ulcer has no known cause in the human body*… **Buruli ulcer patient** (**P: 1)**.

This participant is judging the disease based on the lack of information about the causative organism over the past years.

Another study participant explained Buruli ulcer as;

- *Buruli ulcer is a disease caused by evil spirits… these evil spirits could be in the environment or outside the environment due to the nature of the person affected by the disease*… **Buruli ulcer patient** (**P: 5)**.

Based on the findings, the participant understanding of the disease is that spirits could inflict pain to people in the study setting.

Similarly, another study participant indicated that;

- *Buruli ulcer is when a wound appears in the person and those wounds are very painful*… **Resident without Buruli ulcer patient** (**P: 2)**.

Another study participant remarked as;

- *To me I do not have any knowledge on that [Buruli ulcer] disease and hence there is no proper knowledge of me about the condition…***Resident without Buruli ulcer patient** (**P: 2)**.

This participant appeared to have no knowledge of the disease and could have probable attributed the disease to evil spirits.

#### Participant’s knowledge of signs and symptoms of buruli ulcer

The study participants were asked to indicate their knowledge level on the signs and symptoms of Buruli ulcer in the study setting. The findings are illustrated below.

- *One thing I know about the sign and symptom of Buruli ulcer is that*,* the wound is painful and does not heal for a long time in the person. So those of us [Buruli ulcer patients] with the disease often experience the pain in the night*… **Buruli ulcer patient** (**P: 5)**.

The participant identification of the sign and symptom of Buruli ulcer was linked to the pain they experienced in the night due to the condition.

Another study participant identified the signs and symptoms as;

- *A wound that would just appear in the person on its own without any cause*… **Buruli ulcer patient** (**P: 5)**.

Based on the explanation, study participants identified the sign and symptom of Buruli ulcer linked to the mystery wound in the body that has no cause.

### Theme 2: attitude of residents towards buruli ulcer

The attitude of residents towards Buruli ulcer was examined. Various responses were identified and presented. The attitude of the study participants were presented in two folds. These included the favourable and unfavourable attitudes of the participants towards the disease.

### Favourable attitude of respondents towards the disease

Participants expressed few favourable attitudes towards the disease in these manners.

### Seek support in health centre for buruli ulcer

- *The dressing of the wound is done at the health centre…***Buruli ulcer patient** (**P: 1)**.

The study participants were able to identify there is the need for them to go to the hospital for the wound dressing. This implied that, the participant had a favourable attitude towards the disease.

Another study participant expressed the attitude towards the disease in this manner;

### Manage the condition at home

- *I usually take care of myself with the disease… It is not a good look for some people*… **Buruli ulcer patient** (**P: 5)**.

This participant probably reverted to the use of local medicines to manage the condition at home.

### Unfavourable attitude towards the disease

The unfavourable attitude of the study participants towards the disease are expressed below as;

### Fewer visitations to healthcare to seek healthcare

- *Sometimes they do not visit the healthcare for checking because*,* they wound does not want to heal in the patients*… **Resident without the Buruli ulcer** (**P: 5)**.

The finding from the study suggests how study participants treated and show their attitude towards the disease in the study setting. Therefore, cases were perhaps left to manage the disease themselves at homes.

### Patients are uncomfortable with the disease

- *Sometimes*,* I feel uncomfortable with people who have the Buruli ulcer… so I do not show so much care in the person infected…***Resident without the Buruli ulcer** (**P: 1)**.

The finding suggests that, residents in the study setting had less favourable attitude towards the disease and turn to abandon their relatives with the disease.

#### No concern is given to the disease until it is serious

- *I have been to health centres when the condition was serious*… **Buruli ulcer patient** (**P: 1)**.

The persons living with the Buruli ulcer shown more concern about the disease when the condition was serious among them at the time of the study.

### Less attention to wound due to cost of buying dressing materials

- *Managing the wound is too costly among the cases at the study setting*… **Buruli ulcer patient** (**P: 1)**.

Because the cases were managing the conditions at the study setting, there was less attention to the condition because it was costly to do it. They had to purchase dressing materials for their wounds dressings by themselves.

#### Transportation cost is high to health centre

- *Going to health centre to dress the wound is too costly and expensive…***Buruli ulcer patient** (**P: 1)**.

Persons living with the disease explained that sometimes, it was very difficult to manage the condition because the cost of going to the health centre for dressing was too high and this affected their attitude towards the disease.

#### Less socialization with people in the study setting

- *There is less participation in social activities due to the nature of the disease in me [Buruli ulcer patient]…***Buruli ulcer patient** (**P: 1)**.

The study participants revealed that, there was less participations in social activities due to the nature of the disease. The affected individuals were socially tied down and did not want to be exposed to people due to the stigma associated with the disease.

### Theme 3: practices of residents on buruli ulcer

The practices of residents on Buruli ulcer was examined among the study participants in the study setting. Various responses emerged from the data and illustrated below.

#### Managing buruli ulcer with local herbs

- *I have been using local herbs to manage the disease for some time now… because the hospital medicine is not healing the wound fast*… **Buruli ulcer patient** (**P: 1)**.

This finding from the study provides the argument that residents use the local herbs for the treatment of the disease.

Another study participant remarked as;

- *Here in this place*,* when we see a person with the disease*,* their family members encourage them to seek health by going to the hospital*… **Participant without Buruli ulcer** (**P: 2)**.

The finding provides the opportunity for other members in the family to assist in taking care of Buruli ulcer patients. In the study setting, people with the disease try to seek support from other family members in terms of reaching the hospital or traditional homes or Pastor’s place for care services at the time of the study.

#### Use counter medicine to treat buruli ulcer

- *I sometimes use counter medicines to manage the wound because if the medicine that is given me is finished and I cannot go to the hospital*,* I will use the medicine that I bought to manage the disease*… **Buruli ulcer patient** (**P: 1)**.

The finding indicates that, there was usage of over the counter medicine in the treatment of Buruli ulcer among residents in the study setting at the time of the study.

### Purchase of dressing materials for the wound

- *I have been using dressing materials that I purchased for use in the health centre*… **Buruli ulcer patient** (**P: 1)**.

This means that in the study setting, persons living with the Buruli ulcer had to purchase the dressing materials to be used for the wound dressing when they visited the health centres or hospital for care.

### Visitation of prayer camps for prayers and healing

- *I visit prayer camps for prayers to enable me get the opportunity to be healed*… **Buruli ulcer patient** (**P: 1)**.

In the study setting, persons living with the disease visited prayers camps to ensure that their conditions were improved through divine interventions.

Another participant expressed this as;

- *Buruli ulcer patients do move to attend prayers camps for healing of the wound by Pastors who prophesized that these types of wounds are spiritual and hence need to be healed by divine intervention*… **Participant without Buruli ulcer** (**P: 3)**.

There is a belief that the disease could be cure by religious leaders since most people do not want to place all their hopes in the healthcare medicines provided to them. In the study setting, people were seen at the Prayer camps because that place provided them the opportunity to be able to pray and ask for God’s mercies and healing powers.

### Visit hospital for healthcare

- *I visit the hospital for wound dressing always when I have transportation cost and the dressing materials are available*… **Buruli ulcer patient** (**P: 3)**.

The Buruli ulcer patients visited the hospital to seek healthcare when there was the need at the study setting.

Another participant remarked as;

- *Some of the Buruli ulcer patients visit the clinic for wound dressing once the wound appear in them and have hope that*,* the wound would heal*… **Buruli ulcer patient** (**P: 3)**.

The finding suggests the practice of Buruli ulcer cases going to the hospital to ensure that their condition is better and hence listen to the advice of healthcare professionals who were experts in treating similar cases in the study setting. But the cost of transportation coupled with the belief that, the disease could be treated at the prayer camps affected the nature and pattern of Buruli ulcer cases attending the hospital regularly.

#### Use herbal medicine for managing buruli ulcer

- *The use of herbal medicine to treat the wound is common in this Municipality. The practice is common among the cases [Buruli ulcer cases] who have seen that*,* the hospital medicines that are given to them do not provide immediate relief and healing*… **Buruli ulcer patient** (**P: 4)**.

The belief in the use of herbal medicine often compels people to use it to treat conditions even after they attend healthcare facilities with the same condition.

Another study participant remarked as;

- *The [Buruli ulcer cases] apply the herbal medicines because they believe that they medicines are good for the disease*… **Participant without the Buruli ulcer** (**P: 2)**.

The residents indicated that they used herbal medicine to treat the disease because the herbal medicine was seen and thought to be good among the Buruli ulcer patients.

#### Covering the wound in the person

- *Most of the Buruli ulcer patients cover their wounds well and I think this is okay with them to heal faster…***Participant without Buruli ulcer** (**P: 4)**.

The need for patients to keep their wounds clean and neat to facilitate healing was observed by the study participants at the study setting. This implied that, patients were covering the wounds to ensure that they are well and able to heal faster at the study setting.

## Discussion

The study examines the knowledge, attitude and practices of residents towards Buruli ulcer among residents at the study setting at the time of the study. These respondents were both Buruli ulcer cases and those who were not patients. The study found that those who were cases appeared to explain Buruli ulcer better as compared to those who were not infected with the disease. This finding from the study is similar to the study carried out by [18] where it was found that in Côte D’Ivoire, participants had varied knowledge on Buruli ulcer. The reason in terms of the knowledge difference between Buruli ulcer patients and those without the disease could be linked to patients having more knowledge from the experiences they have acquired in terms of being afflicted by the condition. The residents without the disease knowledge could perhaps be informed by their exposures to those with the disease or learning about it.

The residents had various social constructions in terms of how the disease was manifesting in human bodies. The study found that, majority of the respondents indicated the disease was caused by evil spirits. This finding from the study is similar to that of [19] who also found that in most communities affected by Buruli ulcer in Ghana, witchcraft can be used to inflict others with Buruli ulcer. The implication of this finding is, cases were more likely to delay treatment at health care facilities. The report of the WHO [20] revealed that, the influence of local beliefs on delay in, and access to, appropriate treatment in developing countries was common among Buruli ulcer patients. The study found that, knowledge of respondents varied depending on the educational level of the study participants and other exposures in the study areas. While participants attributed the cause to unknown factor, others said it has no known cause. This finding from the study agrees with [21] where it was found that, participants had varied knowledge in terms of Buruli ulcer causative agent. The study is also similar to [22] where participants had varied knowledge on Buruli ulcer mode of transmission.

The study found that few of the study participants were able to identify possible signs and symptoms of Buruli ulcer. This finding from the study supports [23] where it was found that, participants had knowledge on Buruli ulcer. The finding also supports [18] where in Côte D’Ivoire participants were able to mention signs and symptoms of Buruli ulcer.

The study also found that participants perceived the disease as not serious until it became serious and they now had to visit the healthcare centres. This finding from the study is at variance with [5] where majority of participants perception of Buruli ulcer was good. The study also found that, study participants attributed the cause of the disease to witchcraft and other evil spirits. This finding from the study concurs with [5] where it was found that, participants had poor perception of Buruli ulcer pointing to other supernatural courses. Majority attributed occurrence of Buruli ulcer to witchcraft, spiritual attack, ancestral curse and affliction by *“ofa”* hence the believe that only appeasing the gods, certain sacrifices, traditional healing and deliverances can remedy Buruli ulcer. The study is also similar to [24] where participants thought Buruli ulcer is caused by their own adversaries, including witches as well as [25] study that reported witchcraft as a major cause of the disease perceived by study participants.

In Ghana, sociocultural beliefs and practices strongly influence the health seeking behaviours of people affected by Buruli ulcer. The results showed that some respondents ever visited spiritual centres for God’s protection. This finding from the study agrees with [26] study where similar results were found. This finding from the study also supports [23] where it was suggested that, health education could improve Buruli ulcer patients health seeking behaviours.

The similarity in terms of the results is the role of culture in seeking health care among Ghanaians. The role that cultural factors play in the etiology, explanation, prognosis and treatment seeking behaviour cannot be underscored, because the study provides in depth information on the burden of the disease, the local understanding of the causes of the disease and therefore its management.

The study also found that, some participants were able to give symptoms of the disease. This finding from the study agrees with [27] where it was found that, participants had knowledge of Buruli ulcer. The study also found that Buruli ulcer patients had experienced pain. This finding from the study is similar to [18] where it was found that in Côte D’Ivoire, Buruli ulcer patients had experienced pains. The study also found that, participants had explained the high cost associated with the treatment of Buruli ulcer. This finding from the study agrees with [18] where in Côte D’Ivoire, Buruli ulcer patients had revealed high cost associated with the treatment.

The study also examines the attitude of residents towards the disease. Based on the findings, the study recorded both positive and negative attitude among residents at the study setting. The study found that, residents that were not affected by the disease had negative attitude towards those who had the disease. This finding from the study concurs with [27] where it was found that, participants had negative attitude towards Buruli ulcer patients. The study examines the practices of residents on the disease at the time of the study. Various practices were recorded at the time of the study. Various ways were employed by residents to manage the disease especially among those who were infected. Wound management was one of the practices employed.

The attitude of patients to Buruli ulcer may also lead to secondary microbial infections on the lesions. Most patients reported very late for treatment. Some even worsen the lesion using non-aseptic herbal medications, which are additional source of secondary microbial infections.

Attending hospital is associated with illnesses that are perceived to be caused by natural factors while illnesses that are perceived to have been induced by sorcery need to be addressed by a traditional healer to counteract the sorcery. The implication of this is that patients would delay treatment for the disease in biomedical health facilities by resorting to self and traditional options of treatment. This presents a serious public health concern because when Buruli ulcer advances to a category three stage, it eventually leads to chronic sores and serious deformities leading to disabilities. At category three stages, treatment becomes very costly thereby putting a heavy financial burden on families, health facilities and the nation as a whole.

### Public health education

The findings would provide relevant information that would inform public health education in the Jasikan Municipality. Public health workers in the municipality could use the findings to provide health education to residents in the area to raise awareness about Buruli ulcer. The findings would enhance research in areas relevant to Buruli ulcer in the Oti Region of Ghana.

### Limitations and strength of the study

The study used qualitative approach to conduct the study which limited the sample size. The used of small sample size narrowed the discussions of the study findings around Buruli ulcer in the study. The used of qualitative study made it difficult to generalize the entire findings of the study to other settings in Ghana. The study findings from the participants are subjective in nature. The interviews from the study participants were not also confirmed by participants through focus group discussions. However, the findings were disseminated to major health stakeholders in the study setting. Nonetheless, the study provided the opportunity to explore the knowledge, attitude and practices of residents towards Buruli ulcer in the study setting. The used of qualitative approach could help in the possible generation of hypothesis for a more quantitative study with a large number of study participants in the study setting. The findings would serve as a baseline data in the study setting. The study method could be a limitation.

## Conclusion

The study examined respondents knowledge in terms of how they understood Buruli ulcer. Respondents were found to have been engaged in various practices that helped manage the disease. The visitation of Buruli ulcer cases and their family members to spiritual centres revealed how residents attributed the condition to supernatural causes. The effect is that residents may delay in seeking healthcare and going to prayer camps for divine healing and may latter visit the hospital with the advanced stage of the disease. Public health workers in the municipality could use community centered education, group enlightenment, sensitization, seminars, workshops, mass education and public health awareness campaigns to raise awareness about Buruli ulcer among residents. These measures would help improve residents attitude towards Buruli ulcer, improve residents knowledge on Buruli ulcer and provide enhanced ways residents could improve their practices towards Buruli ulcer.

## Electronic supplementary material

Below is the link to the electronic supplementary material.

Supplementary Material 1

## Data Availability

The dataset is part of the results in the manuscript.

## Abbreviations

IDI In: Depth Interview
REC: Research Ethics Committee
UHAS: University of Health and Allied Sciences
WHO: World Health Organization

## References

[1] Aboagye SY, Kpeli G, Tuffour J, Yeboah-Manu D. (2018). Challenges associated with the treatment of buruli ulcer. J Leukoc Biol. 10.1002/JLB.MR0318-128 30168876

[2] Laso JMG, Mayoral TN, Gutiérrez JJP, González ÉS, Martín BR, Rodríguez LB, Molina JAP. Buruli ulcers in a Spanish aid worker after a stay in Peru. Int J Infect Dis. 2019;78:99–102. 30497990 10.1016/j.ijid.2018.10.012

[3] Garchitorena A, Bonds MH, Guégan JF, Roche B. (2018). Interactions between ecological and socio-economic drivers of buruli ulcer burden in Sub-Saharan africa: opportunities for an improved control. Ecol Evol Infect Diseases: Pathogen Control Public Health Manage Low-Income Ctries, 217.

[4] Simpson, H., Deribe, K., Tabah, E. N., Peters, A., Maman, I., Frimpong, M.,… Cano,J. (2018). Mapping the Global Distribution of Buruli Ulcer Through a Systematic Review with an Evidence Consensus Approach. Available at SSRN 3301628. 10.1016/S2214-109X(19)30171-8 PMC6614043 31200890

[5] Otuh PI, Soyinka FO, Ogunro BN, Akinseye V, Nwezza EE, Iseoluwa-Adelokiki AO, Adeyemo OK. (2018). Perception and incidence of buruli ulcer in Ogun state, South West nigeria: intensive epidemiological survey and public health intervention recommended. Pan Afr Med J, *29*. 10.11604/pamj.2018.29.166.10110 PMC6057600 30050630

[6] Anokye R, Acheampong E, Mprah WK, Sarpong E. Perceived causes and risk factors of buruli ulcer among patients at agogo presbyterian hospital in Ashanti region of Ghana. BMC Res Notes. 2018;11(1):64. 29361986 10.1186/s13104-018-3172-5 PMC5782383

[7] WHO. (2022). Buruli ulcer (Mycobacterium ulcerans infection). 12040740

[8] Kibadi K, Boelaert M, Kayinua M, Minuku JB, Muyembe-Tamfum JJ, Portaels F, Lefèvre P. Therapeutic itineraries of patients with ulcerated forms of *Mycobacterium ulcerans* (Buruli ulcer) disease in rural health zone in the Democratic Republic of congo. Trop Med Int Health. 2009;14(9):1110–6. 19563476 10.1111/j.1365-3156.2009.02324.x

[9] Grietens K, Toomer P, Um Boock E, Hausmann-Muela A, Peeters S, H, Kanobana K. (2012). What role do traditional beliefs play in treatment seeking and delay for buruli ulcer disease? Insights from a mixed methods study in Cameroon. PLoS ONE 7(5), e36954. 10.1371/journal.pone.0036954 PMC3356303 22623964

[10] Stienstra Y, Van Graaft D, Asamoa WT, K., Van Der Werft TS. Beliefs and attitudes towards buruli ulcer in Ghana. Am J Trop Med Hyg. 2002;67(2):207–13. 12389949 10.4269/ajtmh.2002.67.207

[11] District Health Information Management Systems. (2022). Data extracted from the District Health Information Management System 2 (DHIMS 2), April, 2022.

[12] Sangasubana N. How to conduct ethnography research. The Qualitative Research; 2011.

[13] Angrosino M. Doing ethnographic and observational research. Thousand Oaks, CA: Sage; 2007.

[14] Sandelowski M. One is the liveliest number: the case orientation of qualitative research. Res Nurs Health. 1996;19(6):525–9. 8948406 10.1002/(SICI)1098-240X(199612)19:6<525::AID-NUR8>3.0.CO;2-Q

[15] Morse JM. Determining sample size. Qual Health Res. 2000;10(1):3–5.

[16] Green J, Thorogood N. Qualitative methods for health research. London: Sage; 2004.

[17] Francis JJ, Johnston M, Robertson C, Glidewell L, Entwistle V, Eccles MP. What is an adequate sample size? Operationalising data saturation for theory-based interview studies. Psychol Health. 2010;25(10):1229–45. 20204937 10.1080/08870440903194015

[18] Konan, D. O., Mosi, L., Fokou, G., Dassi, C., Narh, C. A., Quaye, C.,… Bonfoh, B.(2019). Buruli ulcer in southern Côte D’Ivoire: dynamic schemes of perception and interpretation of modes of transmission. J Bio Sci. 51(4):520–533. 10.1017/S0021932018000317 30376901

[19] Owusu-Sekyere E. Managing the buruli ulcer morbidity in the Amansie West district of ghana: can Indigenous knowledge succeed? Int J Med Med Sci. 2012;4(9):180–5.

[20] WHO. (2018) *Buruli Ulcer Disease*. URL: http://www.who.int/mediacentre/factsheets/fs199/en/index.html (accessed 25th September 2018).

[21] Vouking M, Tamo Z, V, C, Mbuagbaw L. (2013). The impact of community health workers (CHWs) on Buruli ulcer in sub-Saharan Africa: a systematic review. Pan Afr Med J. 2013;15:19. 10.11604/pamj.2013.15.19.1991 PMC3758852 24009795

[22] Pluschke G, Röltgen K. (2015). Epidemiology and disease burden of Buruli ulcer: a review. Res Rep Trop Med. 2015;(6):59–73.

[23] Gyaase P, Lindsay MK, Acheampong EB, Sampson DB. Knowledge and management of buruli ulcer disease: A case study at Sekyere Afram plains district of Ashanti region, Ghana. Int J Trop DISEASE Health. 2023;44(9):7–21. 10.9734/ijtdh/2023/v44i91428.

[24] Adobea YO, Adamba C. Household perceptions, treatment-seeking behaviors and health outcomes for buruli ulcer disease in a Peri-Urban district in Ghana. Adv Appl Soc. 2012;3:179–86.

[25] Stienstra, Y., Van Der Werf, T. S., Oosterom, E., Nolte, I. M., Van der Graaf, W.T. A., Etuaful, S.,… van der Steege, G. Susceptibility to Buruli ulcer is associated with the SLC11A1 (NRAMP1) D543N polymorphism. Genes & Immunity. 2006;7(3):185–189. 10.1038/sj.gene.6364281 16395392

[26] Adamba C, Owusu AY. Burden of buruli ulcer: how affected households in a Ghanaian district Cope. Afr Study Monogr. 2011;32:1–23.

[27] Akoachere JFK, Nsai FS, Ndip RN. (2016). A community based study on the mode of transmission, prevention and treatment of buruli ulcers in Southwest cameroon: knowledge, attitude and practices. PLoS ONE, 11(5), e0156463. 10.1371/journal.pone.0156463 PMC4881961 27227429

[28] Bayley AC. Buruli ulcer in Ghana. BMJ. 1971;2:401–2. 5575986 10.1136/bmj.2.5758.401-c PMC1795769

[29] Van der Werf TS, van der Graaf WT, Groothuis DG, Knell AJ. Mycobacterium ulcerans infection in Ashanti region, Ghana. Trans R Soc Trop Med Hyg. 1989;83(3):410–3. 2617591 10.1016/0035-9203(89)90521-x

[30] Amofah G, Bonsu F, Tetteh C, Okrah J, Asamoa K, Asiedu K, Addy J. Buruli ulcer in ghana: results of a National case search. Emerg Infect Dis. 2002;8(2):167–70. 11897068 10.3201/eid0802.010119 PMC2732443

