# Supplementary material for: Knowledge, attitude and practices of buruli ulcer among residents in Jasikan municipality of Ghana: an ethnographic study
Source: BMC Public Health. 2025 Jul 3;25:2371. doi: 10.1186/s12889-025-23367-y (PMC12224852; doi:10.1186/s12889-025-23367-y)
Supplement: Supplementary file 1 — Supplementary Material 1 [file 12889_2025_23367_MOESM1_ESM.docx]

**APPENDIX A: INDEPTH INTERVIEW GUIDE**

This in-depth interview guide was designed to enable the study to collect data on Buruli ulcer among residents.

This in-depth interview guide will be designed to enable the study to collect data on Buruli ulcer among residents.

Demographic data of respondents

- How old are you?..........................
- Indicate your sex……………….
- What work do you do?................................
- What is your marital status?...............
- Indicate your educational level?...........................

**Knowledge of respondents on Buruli ulcer**

- What do you know about Buruli ulcer (Sign and symptoms, causes and treatment)?......**Probe for responses**
- What are the signs and symptoms of Buruli ulcer?................................ **Probe for responses**
- What do you know about wound management of Buruli ulcer? -----**Probe for responses**

**Attitude of respondents towards Buruli ulcer**

- How do you feel when people have Buruli ulcer? **Probe for responses**?
- What is your attitude towards people with Buruli ulcer?.... **probe for responses?**

**Practices of respondents on Buruli ulcer**

- What do people in this community do when they are having Buruli ulcer?...**Probe for responses**
- What are some of the local practices that people do when they are having Buruli ulcer?... **Probe for responses**
- What are some of the health related practices people do when they are having Buruli ulcer?..... **Probe for responses**
- Does other family member play a role in any practice of Buruli ulcer treatment?... **Probe for responses**
- Other relevant information worth sharing about Buruli ulcer………………………..

**Thank you**
